# Can Masked Emotion-Laden Words Prime Emotion-Label Words? An ERP Test on the Mediated Account

**DOI:** 10.3389/fpsyg.2021.721783

**Published:** 2021-10-26

**Authors:** Chenggang Wu, Juan Zhang, Zhen Yuan

**Affiliations:** ^1^Multilingual Edu-AI Laboratory, School of Education, Shanghai International Studies University, Shanghai, China; ^2^Faculty of Education, University of Macau, Macau, China; ^3^Centre for Cognitive and Brain Sciences, University of Macau, Macau, China; ^4^Faculty of Health Sciences, University of Macau, Macau, China

**Keywords:** emotion-label words, emotion-laden words, emotion words, ERP, LPC

## Abstract

The present event-related potential (ERP) study explored whether masked emotion-laden words could facilitate the processing of both emotion-label words and emotion-laden words in a valence judgment task. The results revealed that emotion-laden words as primes failed to influence target emotion-label word processing, whereas emotion-laden words facilitated target emotion-laden words in the congruent condition. Specifically, decreased late positivity complex (LPC) was elicited by emotion-laden words primed by emotion-laden words of the same valence than those primed by emotion-laden words of different valence. Nevertheless, no difference was observed for emotion-label words as targets. These findings supported the mediated account that claimed emotion-laden words engendered emotion *via* the mediation of emotion-label words and hypothesized that emotion-laden words could not prime emotion-label words in the masked priming paradigm. Moreover, this study provided additional evidence showing the distinction between emotion-laden words and emotion-label words.

## Introduction

Across various studies of different languages, it has been consistently found that emotion-label words and emotion-laden words differ in a variety of tasks, such as lexical decision task (Kazanas and Altarriba, 2015, 2016a,b; Zhang et al., 2017, 2018a), flanker task (Wu and Zhang, 2019a; Zhang et al., 2019a,b), and affective Simon task (Altarriba and Basnight-Brown, 2011). Emotion-label words (e.g., shame and ecstasy) directly describe an emotional state, whereas emotion-laden words (e.g., butterfly and surgery) indirectly induce emotion *via* elaboration (Sutton and Altarriba, 2016; Zhou and Tse, 2020). Recent event-related potential (ERP) studies found that more substantial brain activation was evoked by emotion-label words than emotion-laden words in both Chinese (Zhang et al., 2017; Wang et al., 2019) and English (Zhang et al., 2018a) in a lexical decision task. For example, Zhang et al. (2018a) found that emotion-label words in English provoked larger N170 than emotion-laden words. There was also research demonstrating that the discrepancy between emotion-laden words and emotion-label words had an impact on perception of emotion (Wu et al., 2019, 2020). Affective picture valence judgment was facilitated by preceding emotion-label words over emotion-laden words with accentuated processing speed and weaker electrophysiological responses, and the facilitation effect was found in both Chinese (Wu et al., 2020) and English (Wu et al., 2019).

Although the separation between emotion-laden words and emotion-label words has received much support (Wu and Zhang, 2019b), it is still unclear how the two kinds of emotion words relate to each other. Emotion-label words and emotion-laden words are by no means irrelevant because when recognizing emotion-laden words, individuals will naturally activate related emotion-label words accordingly (Wu and Zhang, 2019b). For example, when individuals read the word “reward,” they will be reminded of the experiences of receiving a reward and emotions that are embodied in those experiences will also be induced. However, the connection between emotion-label words and emotion-laden words is not one-to-one. Negative emotion-laden words, such as death, will activate various negative emotion concepts, such as fear, sadness, and so on. Therefore, one emotion-laden word can have multiple connections to many emotion-label words, but each of the connections is dependent on situations. The current study employed the masked priming paradigm to examine whether or not masked emotion-laden words could prime emotion-label words.

Investigating the association between emotion-laden words and emotion-label words is of close relevance to the theory explaining the distinction between emotion-label words and emotion-laden words (Knickerbocker, 2014). Altarriba and Basnight-Brown (2011) proposed a mediated account to explain the differences among emotion-label words and emotion-laden words and argued that emotion-laden words could be regarded as a type of “mediated” emotion concepts. Unlike emotion-label words that label emotion concepts straightforwardly, emotion-laden words elicit emotion mediated by the emotion-label words after emotional experiences related to the emotion-laden words are elaborated. Therefore, emotion-label words generated increased emotion activation than emotion-laden words, and this finding has been widely reported (Knickerbocker and Altarriba, 2013; Kazanas and Altarriba, 2015, 2016a,b; Zhang et al., 2017, 2018a, 2019a,b; Wang et al., 2019; Wu et al., 2019, 2020). However, the mediated account claimed by Altarriba and Basnight-Brown (2011) does not specify how emotion-laden words are mediated by emotion-label words. As elucidated before, one emotion-laden word does not activate a single and certain emotion labeled by one emotion-label word. Rather, each emotion-laden word has oblique relationships with emotion activation *via* unpredictable connections to emotion-label words. This individualized and contextualized mapping of emotion-laden words and emotion-label words is an important addition to the original mediated account (Altarriba and Basnight-Brown, 2011).

Extant studies have already provided partial support for the mediated account. Kazanas and Altarriba (2015) examined how emotion-label words and emotion-laden words were different in provoking priming effects in masked and unmasked priming paradigms. In their study, emotion-label words primed emotion-label words, and emotion-laden words primed emotion-laden words. The results revealed a significant priming effect for both emotion-label words and emotion-laden words with the priming effect being larger for emotion-label words, suggesting that emotion-label words and emotion-laden words are distinct categories.

Although Kazanas and Altarriba (2015, 2016a,b) offered much insight into how emotion-label words and emotion-laden words are semantically presented, they did not provide a certain answer to how emotion-laden words and emotion-label words are related to each other. In other words, in their study, when target words were emotion-label words, the primes were also emotion-label words, and the same procedure was applied to emotion-laden words. This operation resulted in one unresolved issue whether emotion-laden words could prime emotion-label words. According to the mediated account (Altarriba and Basnight-Brown, 2011), if emotion-laden words elicit emotion *via* emotion concepts that are labeled by emotion-label words in an individualized and a contextualized manner, it can thus be predicted that emotion-laden words in the masked priming paradigm would not prime emotion-label words as targets. This study attempted to examine whether masked emotion-laden words could prime emotion-label words and test the hypothesis derived from the mediated account (Knickerbocker et al., 2019). As suggested by Knickerbocker et al. (2019), one extension of the study by Kazanas and Altarriba (2015) was to investigate how emotion-label words are primed by emotion-laden words.

One recent event-related potential (ERP) study (Wu et al., 2021) explicitly examined how emotion-label words and emotion-laden words as primes influence target emotion-laden word processing in both masked and unmasked priming paradigms. The overall results confirmed the division of the two types of words, and, more importantly, this distinction could still be observed in the masked condition. Specifically, masked emotion-label words inhibited target emotion-laden words by increasing the error rate and decreasing the processing speed than those target words preceded by masked emotion-laden words. However, one unresolved problem was how emotion-laden words could influence emotion-label words. This study aimed to answer this question according to a previous study (Wu et al., 2021).

In addition, this study measured electrophysiological responses using the ERP technique. One late ERP component (late positivity complex, LPC) related to elaboration during emotion word processing was explored in this study. Emotion-laden words were found to elicit enhanced LPC than emotion-label words, suggesting that processing emotion-laden words is more effortful than emotion-label words (Zhang et al., 2018a). If emotion-label words could be primed by emotion-laden words in the masked condition, it is predicted that emotion-label words would generate a larger LPC when the emotion-label words are preceded by unrelated emotion-laden words (from the different valence) than by related emotion-laden words (from the same valence). If emotion-laden words could not facilitate emotion-label word processing, no modulation on LPC is expected.

## Method

### Participants

About 25 Chinese-English bilingual speakers from the University of Macau were recruited for this study. Due to exceeding artifacts, 5 were excluded, and the remaining 20 Chinese speakers were kept for further processing (3 men, mean age: 27 years). All of the participants were right-handed. They reported that they did not suffer from psychiatric disorders or brain damage. Participants had normal or corrected-to-normal vision. The sample size was determined by calculating the prior power using G^*^power (Faul et al., 2007). Repeated-measure ANOVA requires at least 20 participants when the power is 0.8, and effect size is medium, partial η^2^ = 0.1, also in line with previous studies (Wu and Zhang, 2019a; Zhang et al., 2019a).

### Stimuli

Two sets of Chinese emotion words were selected for the stimuli (Wu et al., 2021). The first set of emotion-laden words that were used as primes was obtained from a recent Chinese norming database (Yao et al., 2017). There were 160 emotion-laden words, including 80 positive words and 80 negative words. Both the negative and positive words were divided into two halves to prime 160 target emotion words. The target words formed the second set of Chinese words, including 80 emotion-label words (40 positive and 40 negative words) and 80 emotion-laden words (40 positive and 40 negative words). The primes were not different among the different conditions in terms of word frequency, strokes, and arousal, all *ps* > 0.05. The same restriction was applied to target words, all *ps* > 0.05 (see Tables 1, 2 for more details). Primes and targets were randomly combined to control the semantic association between the primes and targets. We calculated the word association strength between emotion-label words and emotion-laden words from a recent Chinese word database (Lin et al., 2019) and found no association between the primes and targets (see more details in Discussion).

**Table 1:** Mean and SD in brackets for word characteristics for Chinese emotion-laden words as primes.

	**Emotion-laden words**		**Emotion-laden words**	
	**Primed by label words**		**Primed by laden words**	
	**Negative**	**Positive**	**Negative**	**Positive**
Sample	死囚	花束	火化	春光
	(prisoner)	(flower)	(cremation)	(Spring view)
Word frequency	1.98 (0.79)	1.97 (0.69)	2.14 (0.64)	1.82 (0.75)
Strokes	17.43 (3.94)	18.08 (4.31)	17.70 (4.82)	16.78 (4.86)
Arousal	5.58 (1.36)	5.52 (1.03)	5.43 (1.03)	5.40 (0.91)
Valence	3.49 (1.07)	5.91 (0.63)	3.17 (0.84)	5.98 (0.60)

**Table 2:** Mean and SD in brackets for word characteristics for Chinese emotion-label words and emotion-laden words as targets.

	**Emotion-label words**		**Emotion-laden words**	
	**Negative**	**Positive**	**Negative**	**Positive**
Sample	心疼	舒心	分手	生日
	(sadness)	(joy)	(breakup)	(birthday)
Word frequency	2.37 (0.86)	2.22 (0.84)	2.30 (0.67)	2.37 (0.63)
Strokes	16.78 (4.24)	17.80 (3.31)	16.23 (4.31)	16.55 (5.04)
Arousal	4.78 (0.29)	4.78 (0.47)	4.75 (0.45)	4.88 (0.67)
Valence	2.58 (0.32)	5.29 (0.48)	2.32 (0.57)	5.34 (0.51)

### Procedure

All procedures have been approved by the Institutional Review Board at the University of Macau before the experiment. All participants first signed a consent form before they started the experiment. After setting up the Geodesic Sensor Net (EGI, Eugene, OR, USA), the experimenter described the experimental procedure to participants. At the same time, participants could read the written instruction that was displayed on the monitor at a distance of 70 cm. The task was to determine the valence of the target Chinese emotion words. Each trial started with a 500 ms fixation. A forward mask lasting for 500 ms was followed by a prime word (50 ms). The prime was also masked by a backward mask (10 ms) that was created by overlapping several complex Chinese characters (Zhang et al., 2018b). Afterward, a target emotion word (Song font, 48 points) was presented to participants and disappeared as soon as participants made a response. Each prime was presented two times in two different blocks and was randomly paired with a target word with the same (related condition) or different (unrelated condition) valence. Altogether, 320 trials were dispersed into 8 blocks, each of which included 40 trials. Trials within each block and the blocks were presented randomly. The short rests were inserted between the blocks.

### ERP Recording and Analysis

Scalp voltages were recorded with a 129-channel Geodesic Sensor Net with a sampling rate of 1,000 Hz. The impedance was retained below 50 kΩ during recording. To avoid eyeblinks, at the end of each trial, a notice of allowing eyeblink was displayed for 1,000 ms. The offline data were first filtered with a bandpass of 0.1–30 Hz. The continuous EEG data were further segmented into epochs from 100 ms prior to the presence of the target words. The segmentations were passed through an artifact scan (eyeblink, 70 μV; eye movement, 27.57 μV) and were discarded if the epochs were labeled as an eyeblink or eye movement. The channel was labeled as bad if the change of amplitude exceeded 200 μV. The bad channels were replaced by peripherical sites. However, we deleted the segmentations with more than 10% bad channels. The EEG data were referenced to the average of all electrodes. A baseline correction of −100 to 0 ms on the onset of stimuli was performed. For LPC, three electrodes (Cz, C2, and C4) were chosen during the time window of 500–800 ms. LPC was mostly identified around central sites (Zhang et al., 2018a). We also determine the electrodes using a visual inspection. Moreover, previous studies also found LPC was more salient in the right hemisphere than in the left hemisphere (Zhang et al., 2018a), thus the right central sites were chosen for LPC in this study.

## Results

### Behavioral Results

We deleted the trials that participants made responses slower or faster than Mean ± 2.5 SD for the behavioral data analysis, thereby missing 2.19% data. A three-way ANOVA was conducted. The three within-subject factors were emotion word type, valence, and relatedness.

The behavioral results are summarized in Table 3. Target emotion-label words (0.96) were recognized less accurately than emotion-laden words (0.98), [*F*_(1, 19)_ = 19.343, *p* < 0.001, partial η^2^ = 0.504]. The accuracy rate was higher for positive words (0.98) than for negative words (0.96), [*F*_(1, 19)_ = 9.615, *p* < 0.01, partial η^2^ = 0.336]. There was no significant main effect of relatedness and other interactions in the accuracy rate analysis, *ps* > 0.05. Regarding reaction times, emotion-laden words (697.14 ms) were processed faster than emotion-label words (724.33 ms), [*F*_(1, 19)_ = 10.016, *p* < 0.01, partial η^2^ = 0.345]. In addition, negative words (723.24 ms) were recognized slower than positive words (698.23 ms), [*F*_(1, 19)_ = 7.623, *p* < 0.05, partial η^2^ = 0.286]. No other main effects or interactions were found to reach significance, *ps* > 0.5.

**Table 3:** Mean reaction time (ms) and accuracy rate (%) of emotion-label words and emotion-laden words in brackets as a function of relatedness and valence.

	**Negative**		**Positive**	
	**Related**	**Unrelated**	**Related**	**Unrelated**
Emotion-label	734.17 (95.25)	728.50 (95.63)	720.47 (96.88)	714.17 (96.25)
Emotion-laden	711.53 (95.75)	718.75 (96.75)	669.92 (99.25)	688.37 (98.75)

### ERP Results

**Figure 1:**
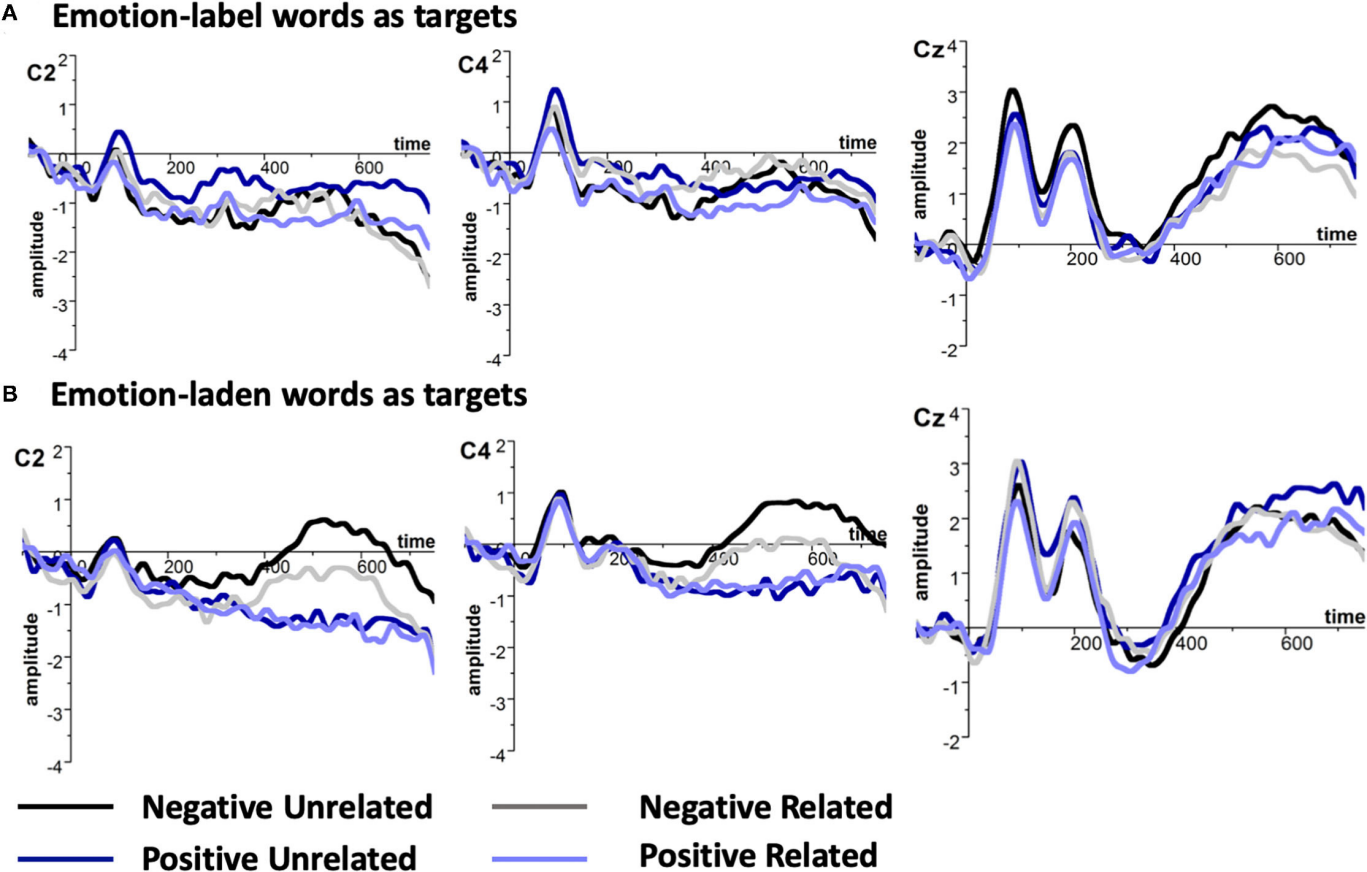
Grand average event-related potentials (ERPs) of late positivity complex (LPC) at selected electrodes **(A)** emotion-label words and **(B)** emotion-laden words as primes, amplitude (μV), and time (ms).

We performed a 2 (Valence: negative and positive) × 2 (Relatedness: related and unrelated) × 2 (Emotion word type: emotion-laden words and emotion-label words) repeated-measure ANOVA. The priming effect was confirmed such that emotion words in the related condition (0.58 μV) provoked a smaller LPC than in the unrelated condition (0.97 μV), [*F*_(1, 19)_ = 5.732, *p* < 0.05, partial η^2^ = 0.232]. To further compare the priming effect between emotion-label words and emotion-laden words, additional ANOVAs containing the two within-subject factors (valence and relatedness) for emotion-label words and emotion-laden words were conducted separately. The result showed that emotion-laden words only produced a priming effect on emotion-laden words, [*F*_(1, 19)_ = 4.585, *p* < 0.05, partial η^2^ = 0.194], rather than on emotion-label words, [*F*_(1, 19)_ = 1.327, *p* > 0.1]. A larger LPC was elicited by the target emotion-laden words that were preceded by the different valence emotion-laden words (1.17 μV) than those that were preceded by the same valence emotion-laden words (0.64 μV). However, no priming effect was found for emotion-label words as targets (0.52 μV in the related condition and 0.77 μV in the unrelated condition). No other main effects or interactions were identified, *ps* > 0.05 (refer to Figure 1).

## Discussion

In this experiment, we investigated whether emotion-laden words as primes could influence target emotion-label and emotion-laden words in the masked priming paradigm. The behavioral results showed the processing differences between the two types of emotion words, replicating many prior examinations (Kazanas and Altarriba, 2015, 2016a,b; Zhang et al., 2017, 2018a, 2019a,b; Wang et al., 2019; Wu and Zhang, 2019a,b; Wu et al., 2019, 2020). Electrophysiological evidence further supported the emotion word-type effect by showing that emotion-laden words rather than emotion-label words could be facilitated by emotion-laden words as masked primes, suggesting that the two categories are emotion-label words and emotion-laden words (Wu and Zhang, 2019b).

This study aimed to examine the mediated account that explained the relationship and the differences between emotion-label words and emotion-laden words (Altarriba and Basnight-Brown, 2011). Altarriba and Basnight-Brown (2011) argued that emotion-laden words are “mediated” by the emotion concepts that are indicated by emotion-label words. Therefore, the emotion activation that is induced by emotion-laden words is achieved after related emotion concepts are activated. For example, a recent study showed that emotion-laden words generated a more substantial electrophysiological activation than emotion-label words, indicating that emotion-laden word processing is more effortful than emotion-label words (Zhang et al., 2018a). However, at a first glance, this study showed contradictory evidence that emotion-laden words had a higher processing speed than emotion-label words with an increased accuracy rate, implying that emotion-label words were harder to recognize in a valence judgment task. This result pattern was, indeed, in line with one recent study that used the emotion flanker task and found that Chinese emotion-laden words were recognized faster than emotion-label words (Zhang et al., 2019b). One difference between the two studies was that there were only 6 emotion-label words in each category in that study (Zhang et al., 2019b), but there were 80 emotion-label words in this study. Therefore, this study, using a large number of emotion words, was an extension of a previous study (Zhang et al., 2019b) by showing that judging the valence of emotion-label words was hard in both flanker and affective priming tasks.

The reason for the difficulty in evaluating the valence of the emotion-label words is that most emotion concepts that are labeled by emotion-label words are not a valence-based representation, especially for negative emotions (e.g., shame, sadness, fear, anger, and boredom). Many researchers (Ekman, 1992; Izard, 2007; Ekman and Cordaro, 2011; Lench et al., 2011) theorized that negative emotions were discrete and negativity was not sufficient to explain the variance between the negative emotions, such as fear and sadness. The ambiguous valence conveyed by emotion-label words was to fill an important void in the mediated account explaining the association between emotion-label words and emotion-laden words. The mediated account claimed that emotion-laden words were mediated by emotion-label words but did not specify how emotion-laden words were mediated by emotion-label words. The emotion-laden words failed to prime emotion-label words, suggesting that emotion-laden words were mediated by emotion-label words in ambiguous associations between the two kinds of emotion words. For example, the word “wedding” can induce many related positive emotions (e.g., happiness and excitement). More importantly, emotions are discrete in both negative and positive categories (Lench et al., 2011; Shiota et al., 2017), increasing the difficulty of judging the valence of target emotion-label words.

It could be argued that word association might influence the priming effect (Hines et al., 1986). We controlled the word association between primes and targets by randomly pairing primes and targets. Therefore, it is assumed that the word association between primes and targets is nearly zero. Based on the Chinese Lexical Association Database (CLAD), one recent Chinese association norming database (Lin et al., 2019), we further analyzed the word association between primes and targets. We retrieved the Baroni-Urbani measure on clauses for each prime emotion word and found that almost all the primes were not associated to targets both in related and unrelated conditions, except a very few words [see the Appendix (Supplementary Material) for the word list and word association, and the two prime words were not found in CLAD]. Further comparisons between primes and targets in related and unrelated for emotion-label words and emotion-laden words found that the word association strength was equally weak for emotion-label words and emotion-laden words as targets, [*F*_(1, 77)_ = 0.737, *p* > 0.39], for emotion word type, [*F*_(1, 77)_ = 1.572, *p* > 0.21], for relatedness, and [*F*_(1, 77)_ = 0.145, *p* > 0.70] for the interaction between emotion word type and relatedness. Based on the restricted word association between primes and targets, it is clear that the priming effect for emotion-laden words was not semantic but affective in essence.

Several limitations of this study should be noted. The first limitation is the definition of emotion-label words and emotion-laden words. It is argued that research on emotion-label words and emotion-laden words is lacking an objective measurement of determining what is an emotion-label word or an emotion-laden word (Hinojosa et al., 2020). One recent normative database of 1,286 Spanish words proffered the ratings of emotional prototypicality that refers to the degree of the typicality of an emotion word (Pérez et al., 2021). The higher prototypicality of an emotion word means that it is more reasonable to be defined as an emotion-label word, such as fear. However, this study did not use this approach to define emotion-label words. Future studies can use the prototype approach to objectively define an emotion-label word. The second limitation is that only adults were included in this study. There is a recent urge to explore how emotional concepts are acquired by children (Hoemann et al., 2019). Therefore, how children process emotion-label words and emotion-laden words would enlighten the emotional development of children across cultures. Future exploration could follow this trend to differentiate emotion-label words and emotion-laden words and examine how emotion-laden words and emotion-label words are associated in mental lexicon of children. The third limitation is that we did not control the concreteness between emotion-label words and emotion-laden words. We retrieved the concreteness of primes and targets from a recent Chinese normative database (Xu and Li, 2020) and found that emotion-label words (48 words were found) were more abstract than emotion-laden words (60 words were found), [*F*_(1, 106)_ = 61.426, *p* < 0.001]. Therefore, the distinction between the two kinds of words can be attributed to the influence of concreteness. However, the primes (emotion-laden words) for target emotion-label words (55 prime words were found) and emotion-laden words (45 prime words were found) were the same on concreteness, [*F*_(1, 98)_ < 1, *p* > 0.47]. The priming effect was found only for emotion-laden word pairs, and this result thus is not related to concreteness. Further research on emotion-label words and emotion-laden words should consider controlling concreteness (Wang et al., 2019). In addition, although we used the masked affective priming paradigm to preclude the strategic influence of processing prime words, future studies can also exploit the unmasked priming paradigm and vary the duration of primes (extending to 1,000 ms) to explore the affective priming of the two types of words (Kazanas and Altarriba, 2016a).

To summarize, the results in this study supported the mediated account, assuming that there was no priming effect of emotion-laden words on emotion-label words. However, the priming effect of emotion-laden words on emotion-laden target words was identified, in line with the mediated account and previous studies (Kazanas and Altarriba, 2015, 2016a,b). Moreover, the distinction between the two kinds of emotion words was also replicated, compatible with the emotion conflict study using the flanker task (Zhang et al., 2019b).

## Data Availability Statement

The datasets generated during and/or analyzed during the current study are available from the corresponding author on reasonable request.

## Ethics Statement

The studies involving human participants were reviewed and approved by Institutional Review Board in the University of Macau. The patients/participants provided their written informed consent to participate in this study.

## Author Contributions

CW and JZ developed the research idea and determined the research design. ZY commented on the research design critically. CW conducted the research, analyzed the data, and drafted the manuscript. JZ and ZY reviewed the manuscript and provided insightful revisions at all stages. All authors have read and agreed to the published version of the manuscript.

## Funding

This study was funded by the University of Macau (MYRG2017-00217-FED, MYRG2016-00193-FED, and MYRG2015-00221-FED). This study was also supported by the Shanghai Philosophy and Social Science Planning Youth Project (2020EYY004), Shanghai Chenguang Talent Program (20CG40), and the Innovative Research Team of Shanghai International Studies University (2020114052).

## Conflict of Interest

The authors declare that the research was conducted in the absence of any commercial or financial relationships that could be construed as a potential conflict of interest.

## Publisher's Note

All claims expressed in this article are solely those of the authors and do not necessarily represent those of their affiliated organizations, or those of the publisher, the editors and the reviewers. Any product that may be evaluated in this article, or claim that may be made by its manufacturer, is not guaranteed or endorsed by the publisher.
