# Supplementary material for: Can Masked Emotion-Laden Words Prime Emotion-Label Words? An ERP Test on the Mediated Account
Source: Front Psychol. 2021 Oct 26;12:721783. doi: 10.3389/fpsyg.2021.721783 (PMC8576488; doi:10.3389/fpsyg.2021.721783)
Supplement: Supplementary file 1 [file Table_1.DOCX]

Appendix: Primes and targets and their association strength

| Emotion-laden words prime emotion-laden words | | | | | | |
| --- | --- | --- | --- | --- | --- | --- |
|  | Related | | | Unrelated | | |
|  | Prime | Target | Association | Prime | Target | Association |
| 1 | 成就 | 拥抱 | 0 | 误会 | 拥抱 | 0.5 |
| 2 | 学识 | 嫁妆 | 0 | 倒塌 | 嫁妆 | 0 |
| 3 | 宝刀 | 樱花 | 0 | 炮灰 | 樱花 | 0 |
| 4 | 战友 | 美女 | 0 | 击毙 | 美女 | 0 |
| 5 | 楷模 | 春雨 | 0 | 呕吐 | 春雨 | 0 |
| 6 | 表扬 | 佳丽 | 0 | 贫民 | 佳丽 | 0 |
| 7 | 礼炮 | 阳光 | 0 | 遗骸 | 阳光 | 0 |
| 8 | 兄弟 | 营养 | 0 | 草包 | 营养 | 0 |
| 9 | 贵人 | 生日 | 0 | 手铐 | 生日 | 0 |
| 10 | 同胞 | 赞赏 | 0 | 诽谤 | 赞赏 | 0 |
| 11 | 名声 | 星空 | 0 | 暴雨 | 星空 | 0 |
| 12 | 硬币 | 校园 | 0 | 囚禁 | 校园 | 0 |
| 13 | 素质 | 笑脸 | 0 | 忽略 | 笑脸 | 0 |
| 14 | 首创 | 使命 | 0 | 洪水 | 使命 | 0 |
| 15 | 糕点 | 丰收 | 0 | 荒岛 | 丰收 | 0 |
| 16 | 白鸽 | 温泉 | 0 | 墓碑 | 温泉 | 0 |
| 17 | 茁壮 | 友谊 | 0 | 草率 | 友谊 | 0 |
| 18 | 熊猫 | 智慧 | 0 | 刀痕 | 智慧 | 0 |
| 19 | 经典 | 年轻 | 0.35 | 烟灰 | 年轻 | N/A |
| 20 | 前景 | 正义 | 0 | 鬼魂 | 正义 | 0 |
| 21 | 条理 | 皇冠 | 0 | 去世 | 皇冠 | 0 |
| 22 | 恩师 | 利润 | 0 | 疯子 | 利润 | 0 |
| 23 | 晚霞 | 赞美 | 0 | 烧伤 | 赞美 | 0 |
| 24 | 凯旋 | 金牌 | 0 | 荒漠 | 金牌 | 0 |
| 25 | 马戏 | 玫瑰 | 0 | 溃疡 | 玫瑰 | 0 |
| 26 | 宝藏 | 知己 | 0 | 废品 | 知己 | 0 |
| 27 | 奖状 | 黄金 | 0 | 偏见 | 黄金 | 0 |
| 28 | 奖章 | 女神 | 0 | 拐杖 | 女神 | 0 |
| 29 | 养生 | 圣洁 | 0 | 火化 | 圣洁 | 0 |
| 30 | 勇士 | 宝石 | 0 | 腐蚀 | 宝石 | 0 |
| 31 | 礼花 | 生长 | N/A | 背叛 | 生长 | 0 |
| 32 | 合影 | 亲友 | 0.49 | 地震 | 亲友 | 0.36 |
| 33 | 人才 | 仁爱 | 0 | 诬告 | 仁爱 | 0 |
| 34 | 喜鹊 | 恋人 | 0 | 轰炸 | 恋人 | 0 |
| 35 | 果冻 | 信念 | 0 | 恶魔 | 信念 | 0 |
| 36 | 春光 | 亲吻 | 0 | 鼻涕 | 亲吻 | 0 |
| 37 | 剪彩 | 喝彩 | 0 | 蚜虫 | 喝彩 | 0 |
| 38 | 娶亲 | 冠军 | 0 | 囚徒 | 冠军 | 0 |
| 39 | 贵族 | 愿望 | 0 | 尸骨 | 愿望 | 0 |
| 40 | 佳肴 | 红包 | 0 | 病房 | 红包 | 0 |
| 41 | 误会 | 垃圾 | 0.27 | 成就 | 垃圾 | 0 |
| 42 | 倒塌 | 诈骗 | 0 | 学识 | 诈骗 | 0 |
| 43 | 炮灰 | 妓女 | 0 | 宝刀 | 妓女 | 0 |
| 44 | 击毙 | 土匪 | 0 | 战友 | 土匪 | 0 |
| 45 | 呕吐 | 耻辱 | 0 | 楷模 | 耻辱 | 0 |
| 46 | 贫民 | 伤痕 | 0 | 表扬 | 伤痕 | 0 |
| 47 | 遗骸 | 棺材 | 0 | 礼炮 | 棺材 | 0 |
| 48 | 草包 | 地震 | 0 | 兄弟 | 地震 | 0 |
| 49 | 手铐 | 奸商 | 0 | 贵人 | 奸商 | 0 |
| 50 | 诽谤 | 淫秽 | 0 | 同胞 | 淫秽 | 0 |
| 51 | 暴雨 | 关押 | 0 | 名声 | 关押 | 0 |
| 52 | 囚禁 | 坠机 | 0 | 硬币 | 坠机 | 0 |
| 53 | 忽略 | 凶手 | 0 | 素质 | 凶手 | 0 |
| 54 | 洪水 | 拐卖 | 0 | 首创 | 拐卖 | 0 |
| 55 | 荒岛 | 暗算 | 0 | 糕点 | 暗算 | 0 |
| 56 | 墓碑 | 分手 | 0 | 白鸽 | 分手 | 0 |
| 57 | 草率 | 乞丐 | 0 | 茁壮 | 乞丐 | 0 |
| 58 | 刀痕 | 牢狱 | 0 | 熊猫 | 牢狱 | 0 |
| 59 | 烟灰 | 焚烧 | N/A | 经典 | 焚烧 | 0 |
| 60 | 鬼魂 | 血迹 | 0 | 前景 | 血迹 | 0 |
| 61 | 去世 | 烈日 | 0 | 条理 | 烈日 | 0 |
| 62 | 疯子 | 骗子 | 0.73 | 恩师 | 骗子 | 0 |
| 63 | 烧伤 | 败类 | 0 | 晚霞 | 败类 | 0 |
| 64 | 荒漠 | 疤痕 | 0 | 凯旋 | 疤痕 | 0 |
| 65 | 溃疡 | 坎坷 | 0 | 马戏 | 坎坷 | 0 |
| 66 | 废品 | 毒贩 | 0 | 宝藏 | 毒贩 | 0 |
| 67 | 偏见 | 哽咽 | 0 | 奖状 | 哽咽 | 0 |
| 68 | 拐杖 | 残骸 | 0 | 奖章 | 残骸 | 0 |
| 69 | 火化 | 枪杀 | 0 | 养生 | 枪杀 | 0 |
| 70 | 腐蚀 | 堵车 | 0 | 勇士 | 堵车 | 0 |
| 71 | 背叛 | 肮脏 | 0 | 礼花 | 肮脏 | NA |
| 72 | 地震 | 屠杀 | 0 | 合影 | 屠杀 | 0 |
| 73 | 诬告 | 仇人 | 0 | 人才 | 仇人 | 0 |
| 74 | 轰炸 | 叛徒 | 0 | 喜鹊 | 叛徒 | 0 |
| 75 | 恶魔 | 空难 | 0 | 果冻 | 空难 | 0 |
| 76 | 鼻涕 | 出轨 | 0 | 春光 | 出轨 | 0 |
| 77 | 蚜虫 | 绑架 | 0 | 剪彩 | 绑架 | 0 |
| 78 | 囚徒 | 僵尸 | 0 | 娶亲 | 僵尸 | 0 |
| 79 | 尸骨 | 枯萎 | 0 | 贵族 | 枯萎 | 0 |
| 80 | 病房 | 毒蛇 | 0 | 佳肴 | 毒蛇 | 0 |
| Emotion-laden words prime emotion-label words | | | | | | |
|  | Related | | | Unrelated | | |
|  | Prime | Target | Association | Prime | Target | Association |
| 1 | 水晶 | 沉醉 | 0 | 拾荒 | 沉醉 | 0 |
| 2 | 淑女 | 满意 | 0 | 囚衣 | 满意 | 0 |
| 3 | 月亮 | 心醉 | 0 | 车祸 | 心醉 | 0 |
| 4 | 旅游 | 乐意 | 0.22 | 咆哮 | 乐意 | 0 |
| 5 | 烟花 | 期待 | 0 | 事故 | 期待 | 0 |
| 6 | 欢呼 | 安然 | 0 | 出殡 | 安然 | 0 |
| 7 | 翡翠 | 宽心 | 0 | 脏物 | 宽心 | 0 |
| 8 | 火炬 | 愉快 | 0 | 瘫痪 | 愉快 | 0 |
| 9 | 奖券 | 庆幸 | 0 | 花圈 | 庆幸 | 0 |
| 10 | 典礼 | 如意 | 0 | 死囚 | 如意 | 0 |
| 11 | 款待 | 入迷 | 0 | 血迹 | 入迷 | 0 |
| 12 | 学问 | 狂喜 | 0 | 糊涂 | 狂喜 | 0 |
| 13 | 铃铛 | 安宁 | 0 | 衰老 | 安宁 | 0 |
| 14 | 风韵 | 舒服 | 0 | 谩骂 | 舒服 | 0 |
| 15 | 事业 | 迷恋 | 0 | 污垢 | 迷恋 | 0 |
| 16 | 榜样 | 喜欢 | 0 | 穷酸 | 喜欢 | 0 |
| 17 | 花园 | 甜美 | 0.36 | 烈火 | 甜美 | 0 |
| 18 | 嫩绿 | 怡然 | 0 | 困倦 | 怡然 | 0 |
| 19 | 花坛 | 满足 | 0 | 疯狗 | 满足 | 0 |
| 20 | 流星 | 如愿 | 0 | 失眠 | 如愿 | 0 |
| 21 | 炸弹 | 失望 | 0 | 繁华 | 失望 | 0 |
| 22 | 责备 | 慌乱 | 0 | 楷模 | 慌乱 | 0 |
| 23 | 囚衣 | 恼怒 | 0 | 淑女 | 恼怒 | 0 |
| 24 | 咆哮 | 忧虑 | 0 | 旅游 | 忧虑 | 0 |
| 25 | 拐骗 | 丢脸 | 0 | 国旗 | 丢脸 | 0 |
| 26 | 瘫痪 | 慌张 | 0 | 火炬 | 慌张 | 0 |
| 27 | 死囚 | 低落 | 0 | 典礼 | 低落 | 0 |
| 28 | 血迹 | 烦闷 | 0 | 款待 | 烦闷 | 0 |
| 29 | 衰老 | 害怕 | 0 | 铃铛 | 害怕 | 0 |
| 30 | 谩骂 | 愤恨 | 0 | 风韵 | 愤恨 | 0 |
| 31 | 处分 | 心酸 | 0 | 老乡 | 心酸 | 0 |
| 32 | 排斥 | 悲伤 | 0 | 收养 | 悲伤 | 0 |
| 33 | 疯狗 | 寂寞 | 0 | 花坛 | 寂寞 | 0 |
| 34 | 死刑 | 抑郁 | 0 | 重逢 | 抑郁 | 0 |
| 35 | 战俘 | 愤怒 | 0 | 旺盛 | 愤怒 | 0 |
| 36 | 包庇 | 失落 | 0 | 粮食 | 失落 | 0 |
| 37 | 敌人 | 为难 | 0 | 浪花 | 为难 | 0 |
| 38 | 杀戮 | 恼火 | 0 | 寿星 | 恼火 | 0 |
| 39 | 失眠 | 生气 | 0.4 | 流星 | 生气 | 0 |
| 40 | 报复 | 讨厌 | 0 | 天鹅 | 讨厌 | 0 |
| 41 | 绸缎 | 向往 | 0 | 拥挤 | 向往 | 0 |
| 42 | 繁华 | 顺畅 | 0 | 炸弹 | 顺畅 | 0 |
| 43 | 楷模 | 享受 | 0 | 责备 | 享受 | 0 |
| 44 | 国旗 | 愉悦 | 0 | 拐骗 | 愉悦 | 0 |
| 45 | 榕树 | 舒畅 | 0 | 宦官 | 舒畅 | 0 |
| 46 | 老乡 | 痛快 | 0 | 处分 | 痛快 | 0 |
| 47 | 花束 | 从容 | 0 | 灰烬 | 从容 | 0 |
| 48 | 漫画 | 快活 | 0 | 昏迷 | 快活 | 0 |
| 49 | 收养 | 舒坦 | 0 | 排斥 | 舒坦 | 0 |
| 50 | 风景 | 欢快 | 0 | 鬼混 | 欢快 | 0 |
| 51 | 倾听 | 着迷 | 0 | 牢笼 | 着迷 | 0 |
| 52 | 重逢 | 自豪 | 0 | 死刑 | 自豪 | 0 |
| 53 | 旺盛 | 安定 | 0 | 战俘 | 安定 | 0 |
| 54 | 珠宝 | 快慰 | 0 | 陵墓 | 快慰 | 0 |
| 55 | 粮食 | 顺心 | 0 | 包庇 | 顺心 | 0 |
| 56 | 渊博 | 欣喜 | 0 | 贿赂 | 欣喜 | 0 |
| 57 | 浪花 | 欢笑 | 0 | 敌人 | 欢笑 | 0 |
| 58 | 寿星 | 舒心 | 0 | 杀戮 | 舒心 | 0 |
| 59 | 嘉宾 | 好奇 | 0 | 溃烂 | 好奇 | 0 |
| 60 | 天鹅 | 爽快 | 0 | 报复 | 爽快 | 0 |
| 61 | 拥挤 | 沮丧 | 0 | 绸缎 | 沮丧 | 0 |
| 62 | 拾荒 | 惧怕 | 0 | 水晶 | 惧怕 | 0 |
| 63 | 车祸 | 恐惧 | 0 | 月亮 | 恐惧 | 0 |
| 64 | 事故 | 压抑 | 0 | 烟花 | 压抑 | 0 |
| 65 | 出殡 | 丢人 | 0 | 欢呼 | 丢人 | 0 |
| 66 | 脏物 | 心烦 | 0 | 翡翠 | 心烦 | 0 |
| 67 | 花圈 | 痛心 | 0 | 奖券 | 痛心 | 0 |
| 68 | 宦官 | 担心 | 0 | 榕树 | 担心 | 0.19 |
| 69 | 糊涂 | 烦躁 | 0 | 学问 | 烦躁 | 0 |
| 70 | 污垢 | 伤心 | 0 | 事业 | 伤心 | 0.27 |
| 71 | 穷酸 | 愁苦 | 0 | 榜样 | 愁苦 | 0 |
| 72 | 灰烬 | 愤慨 | 0 | 花束 | 愤慨 | 0 |
| 73 | 昏迷 | 心疼 | 0 | 漫画 | 心疼 | 0 |
| 74 | 烈火 | 哀伤 | 0 | 花园 | 哀伤 | 0 |
| 75 | 困倦 | 发慌 | 0 | 嫩绿 | 发慌 | 0 |
| 76 | 鬼混 | 忧郁 | 0 | 风景 | 忧郁 | 0 |
| 77 | 牢笼 | 嫉妒 | 0 | 倾听 | 嫉妒 | 0 |
| 78 | 陵墓 | 郁闷 | 0 | 珠宝 | 郁闷 | 0 |
| 79 | 贿赂 | 难过 | 0 | 渊博 | 难过 | 0 |
| 80 | 溃烂 | 厌烦 | 0 | 嘉宾 | 厌烦 | 0 |
